# Linker Engineering in Stapled Peptides for Enhanced Membrane Permeability: Screening and Optimization Strategies

**DOI:** 10.3390/ijms27073077

**Published:** 2026-03-27

**Authors:** Min Zhao, Baojian Li, Ying Gao, Rui Zhang, Subinur Ahmattohti, Jie Li, Xinbo Shi

**Affiliations:** 1 School of Pharmacy, Xinjiang Second Medical College, Karamay 834000, China; 18232189381@163.com (M.Z.); libaojian@xjsmc.edu.cn (B.L.); 18829190996@163.com (Y.G.); zhangrui@xjsmc.edu.cn (R.Z.); 18016910278@163.com (S.A.); 2 Science and Innovation Center, Xinjiang Second Medical College, Karamay 834000, China; 3 Shaanxi Collaborative Innovation Center of Chinese Medicine Resources Industrialization, Shaanxi University of Chinese Medicine, Xianyang 712046, China

**Keywords:** stapled peptides, linker engineering, membrane permeability, high-throughput screening (HTS), intramolecular hydrogen bond (IMHB), chameleonicity, intracellular delivery, rational design

## Abstract

The optimization of membrane permeability is a pivotal approach for mitigating late-stage failures in peptide drug development. By leveraging linker chemical diversity, stapled peptides utilize linker engineering to precisely modulate key physicochemical parameters—such as lipophilicity and conformational constraints—to overcome the desolvation energy penalty. This review systematically evaluates linker-based strategies for enhancing the permeability of stapled peptides, categorized into two primary dimensions: (1) high-throughput screening (HTS) compatibility, focusing on the integration of functionalized linkers into mRNA display, phage display, and DNA-encoded libraries (DELs) to identify lead scaffolds with inherent permeability potential during early discovery; and (2) post-screening structural refinement, covering rational design strategies including intramolecular hydrogen-bond (IMHB) shielding, “chameleonic” adaptations, and stimuli-responsive reversible stapling. Furthermore, we analyze the paradigm shift in assessment methodologies from qualitative imaging to quantitative cytosolic delivery assays, which have deepened our understanding of mechanisms such as the charge/lipophilicity threshold balance and metabolism-driven trapping. Overall, linker engineering provides a robust technical roadmap for developing the next generation of cell-permeable stapled peptide therapeutics.

## 1. Introduction

Within the extensive scope of contemporary drug development, the modulation of protein–protein interactions (PPIs) stands as one of the most challenging and high-potential frontiers [1,2]. The topographical nature of PPI interfaces—typically expansive, flat, and devoid of deep hydrophobic pockets—precludes the effective binding of conventional small molecules that comply with the “Rule of Five” [3,4]. Conversely, while macromolecular biologics like monoclonal antibodies offer exquisite target specificity, their clinical utility is fundamentally limited to the extracellular space by their inability to traverse the plasma membrane [5]. Peptides, by virtue of their distinctive molecular weight, exceptional selectivity, and expansive chemical space, have emerged as a compelling ‘middle-ground’ therapeutic modality.

Despite their therapeutic promise, the transition of linear peptide sequences into viable drug candidates is frequently thwarted by fundamental physicochemical barriers [6,7,8]. Natural peptides are inherently rich in polar backbone amides—serving as both hydrogen-bond donors and acceptors—and charged side chains. The energetic cost for these polar groups to shed aqueous solvation shells and partition into the hydrophobic lipid core, known as the “desolvation penalty”, is prohibitively high [9]. Furthermore, linear peptides exist as high-entropy conformational ensembles in solution; constraining these flexible chains into specific, membrane-permeable conformations necessitates a significant entropic sacrifice, further impeding effective translocation [10].

To mitigate these limitations, researchers, inspired by natural structures such as Cyclosporin A, have extensively investigated *N*-methylation and related backbone modifications [11]. Although reducing the number of hydrogen-bond donors (HBDs) by substituting amide hydrogens can lower the energetic threshold for membrane entry, this strategy inevitably creates a critical trade-off: the disruption of internal hydrogen-bonding networks increases conformational flexibility. This added entropy frequently results in a precipitous drop in binding affinity, as the peptide fails to maintain the rigid, bioactive conformation required for target recognition [12,13,14,15].

Beyond these backbone modifications, macrocyclization—exemplified by head-to-tail and disulfide-bridged architectures—provides a more robust and systemic approach to surmounting the barriers of peptide membrane permeability [16,17]. By not only pre-organizing peptides into rigid conformations and masking solvent-exposed charged termini to minimize conformational entropy loss and metabolic degradation but also harnessing the “chameleonic property,” macrocyclization effectively facilitates the dynamic sequestration of residual polar groups from the environment. Far from a static masking state, true chameleonicity represents a solvent-dependent conformational plasticity. A quintessential example is Cyclosporin A (CsA), which adopts a hairpin-like conformation in non-polar lipid membranes—forming intramolecular hydrogen bonds (IMHBs) to reduce solvent-exposed HBDs—yet undergoes a significant shift in polar media to expose these donors for target binding [18]. This transition results in a profound 3D polar surface area (PSA) difference, enabling passive permeability far exceeding Lipinski’s “Rule of Five” [19].

**Figure 1:**
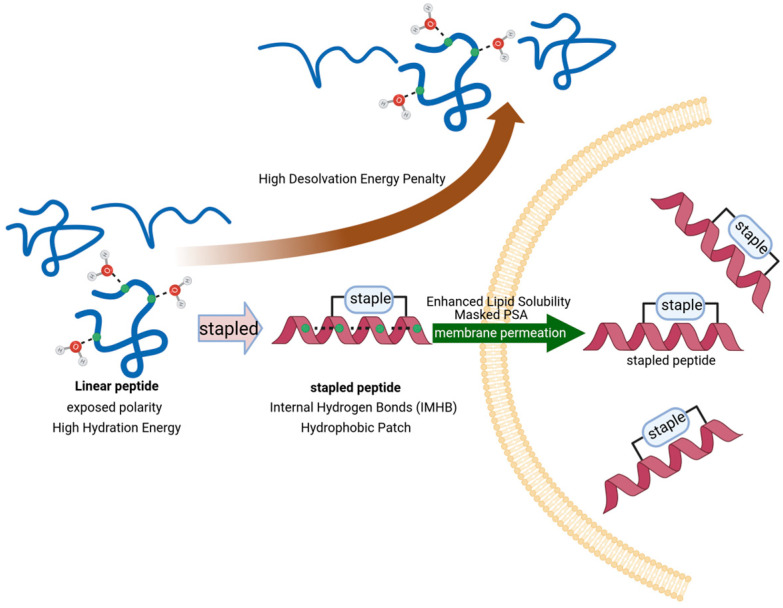
Biophysical mechanism of linker-mediated enhancement of peptide membrane permeability. Linear peptides exist as high-entropy random coils with high hydration energy due to solvent-exposed polar amides, resulting in a significant desolvation energy penalty during membrane translocation. The introduction of a chemical staple induces conformational pre-organization into a stable α-helix, facilitating the formation of IMHB networks and hydrophobic patches. This synergy effectively masks the PSA and enhances lipid solubility, thereby promoting efficient membrane translocation.

As an advanced iteration of macrocyclization technology, peptide stapling primarily involves the introduction of chemical crosslinks at the *i*, *i* + 4 (spanning one helical turn) or *i*, *i* + 7 (spanning two helical turns) positions to precisely induce and stabilize α-helical conformations. In recent years, a plethora of reviews has emerged focusing on their synthetic strategies, screening methodologies, and structural optimizations [20,21]. As illustrated in Figure 1, traditional perspectives hold that such robust conformational constraints not only markedly enhance the binding affinity of peptides toward target proteins but, more crucially, facilitate the physical shielding of polar backbone amides by reinforcing helicity [22]. Concurrently, the intrinsic physicochemical properties of the linkers—such as the introduction of hydrophobic moieties—play an indispensable role in modulating overall molecular polarity and strengthening interactions with lipid bilayers [23]. Nevertheless, despite the successive elucidation of various peptide transmembrane mechanisms, a systematic review focusing on strategies to optimize the membrane permeability of stapled peptides remains overdue.

Against this backdrop, this review provides a comprehensive overview of the pivotal role of linker engineering in optimizing peptide membrane permeability. The content is organized around two core dimensions: first, it discusses the integration of functionalized linkers into high-throughput display platforms—such as mRNA display, phage display, and DELs—to facilitate the “front-loading” of permeability traits during the early selection of macrocyclic leads. Second, it provides an in-depth analysis of sophisticated chemical modification strategies (e.g., leveraging chameleonicity, intramolecular hydrogen bonding, and charge balancing) designed to enhance both permeability and drug-like properties. Additionally, the emerging role of computational modeling and machine learning in streamlining permeability prediction is briefly discussed to provide a future perspective. Furthermore, by incorporating recent advancements in permeability assays, this review systematically elucidates the molecular mechanisms underlying the transmembrane transport of stapled peptides. By bridging the gap between high-throughput discovery and rational post-screening refinement, this work aims to provide a robust technical roadmap and theoretical framework for developing next-generation peptide therapeutics against challenging “undruggable” intracellular targets.

## 2. Linkers Derived from In Vitro High-Throughput Screening

### 2.1. Arylene-Based Linkers via Thiol-Mediated Nucleophilic Substitution

To rapidly explore the vast chemical space of stapled peptides, in vitro HTS platforms—including mRNA display, phage display, and DELs—have become indispensable tools [24,25,26,27,28,29,30,31]. Unlike traditional rational design, these platforms allow for the simultaneous evaluation of billions of peptide sequences. However, the success of such screenings relies heavily on the choice of linkers that are not only chemically robust but also compatible with aqueous, biocompatible conditions. Consequently, functionalized linkers that can be incorporated via efficient, bioorthogonal chemistries have emerged as the “bridge” connecting high-capacity selection with optimized pharmacological properties. The thiol group of cysteine, with its high selectivity and nucleophilicity, can readily undergo *S*_N_2 and *S*_N_Ar reactions under biocompatible conditions, thus enabling efficient reactions with benzyl bromides and polyfluoroarenes, which has attracted significant attention.

#### 2.1.1. Aryl Linkers: Conformational Rigidification

Initially, all-hydrocarbon stapled peptides strongly demonstrated the contribution of conformational locking to the enhancement of cellular membrane permeability by stabilizing the α-helical structure [32]. However, ruthenium-catalyzed ring-closing metathesis (RCM), the core reaction on which they rely, exhibits poor compatibility with aqueous biological environments, rendering it incompatible with HTS platforms such as in vitro display systems. In contrast, although disulfide bonds possess excellent aqueous compatibility, their inherent instability in the reductive intracellular microenvironment severely limits their utility for long-term intracellular targeting applications [33].

**Figure 2:**
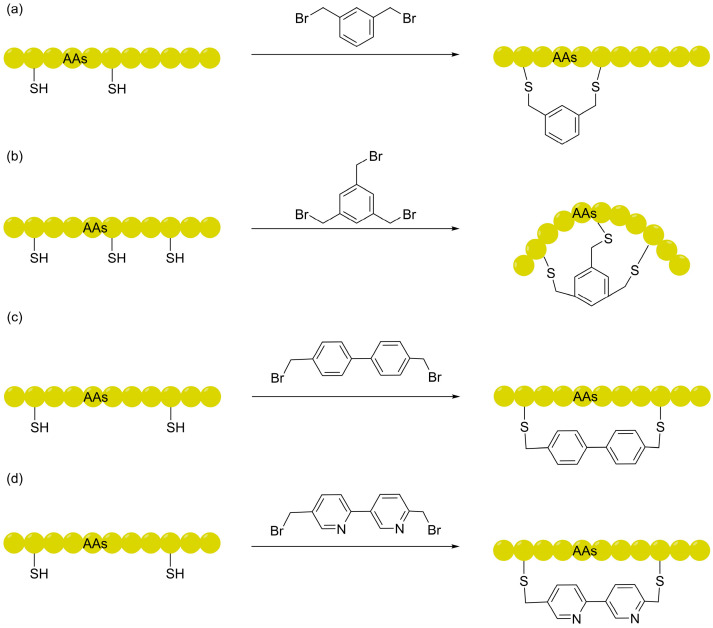
Chemical strategies for the construction of stapled and multicyclic peptides using aromatic linkers: (**a**) CLIPS technology utilizing mDBMB for monocyclization; (**b**) biphenyl-based linkers for hydrophobic shielding; (**c**) construction of bicyclic architectures via C_3_-symmetric TBMB; (**d**) bipyridine (BPy) linkers for backbone shielding via intramolecular hydrogen bonding.

Against this backdrop, the CLIPS (Chemical Linkage of Peptides onto Scaffolds) technology introduced by Timmerman et al. spurred the development of aryl linkers, exemplified by *m*-xylene (mDBMB) [34]. By leveraging the *S*_N_2 nucleophilic substitution of cysteine residues under mild, biocompatible conditions, this strategy establishes chemically robust aryl-thioether bridges. This approach not only fundamentally mitigates the risks of instability inherent to traditional disulfide bonds within the intracellular reductive microenvironment but also offers a dependable chemical anchor for the precise conformational steering of the peptide backbone [24]. Although initial investigations primarily highlighted phenotypic improvements in proteolytic stability and target binding affinity, deeper biophysical scrutiny suggests that this α-helical constraint—analogous to that of all-hydrocarbon linkers—serves as the mechanistic cornerstone for traversing cellular barriers. By anchoring the peptide in its bioactive state through potent pre-organization, the significant entropic penalty typically incurred by conformational reorganization during translocation across the hydrophobic phospholipid bilayer is substantially diminished. This mechanism of “energetic pre-compensation” via structural rigidification provides a robust theoretical framework for the design and evolution of stapled peptide architectures with superior cell-penetrating potential [35]. Representative chemical strategies for the construction of such membrane-permeable architectures—ranging from simple CLIPS-based monocyclization to complex bicyclic and backbone-shielding scaffolds—are summarized in Figure 2.

As screening technologies iterated, linker design evolved toward a strategic emphasis on hydrophobic shielding. The Qing Lin group demonstrated that biphenyl-based linkers, by providing an expanded hydrophobic patch, not only enhance binding affinity but also effectively compensate for the desolvation penalty of the peptide backbone [36]. Building on these monocyclic aryl linkers, the application of 1,3,5-tris(bromomethyl)benzene (TBMB), pioneered by Heinis and Winter, marked a significant evolution toward ultra-rigid bicyclic architectures [37]. Owing to its C_3_-symmetric scaffold and efficient *S*_N_2 reactivity, TBMB has established itself as a foundational tool for constructing vast libraries, particularly in phage display platforms pioneered by Heinis and Winter, as well as emerging mRNA display systems [38]. From a biophysical perspective, this “topological condensation” significantly compresses the molecular radius of gyration, minimizing the entropic penalty during target binding and conferring exceptional proteolytic stability [39]. Utilizing this architecture, the Slavoff group developed the TBMB-linked bicyclic peptide CP21. Although its basal membrane permeability is relatively weak, its extraordinary target affinity allowed for successful intracellular engagement, as validated by CETSA. By bypassing genetic compensation, this chemical probe identified 76 novel DCP2 substrates, highlighting the potential of such scaffolds to target “undruggable” intracellular PPIs [40]. Furthermore, recent explorations of 1,2,3-TBMB isomers by the Lau group have further expanded the conformational landscape of macrocyclic peptides, driving the screening paradigm from “coarse-grained rigidification” toward “precision-tailored geometric topology” [41].

#### 2.1.2. Perfluoroaromatic Linkers: Leveraging the “Fluorine Effect” for Translocation

**Figure 3:**
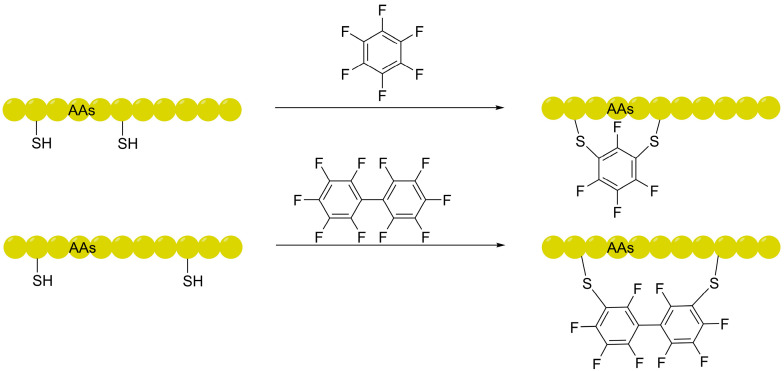
Schematic representation of the *S*_N_Ar reaction between cysteine thiols. This strategy utilizes the “fluorine effect” to increase overall lipophilicity and facilitate membrane translocation, establishing a foundational framework for the subsequent development of advanced fluorinated shuttles.

Parallel to advancements in all-hydrocarbon and alkyl-aryl stapling technologies, the integration of perfluoroaromatic linkers represents a paradigm shift in leveraging the unique “fluorine effect” to enhance membrane permeability [42,43,44,45]. Pioneered by the Pentelute group, this strategy exhibits high selectivity for cysteine, in contrast to the limitation of bromoalkyl/benzyl chemistry that tends to produce over-alkylation products. Its core lies in constructing stable aryl thioether bonds via *S*_N_Ar between cysteine thiols and electron-deficient perfluoroarenes (e.g., hexafluorobenzene, decafluorobiphenyl); flow cytometry and confocal microscopy have confirmed that perfluoroaryl-stapled peptides possess significantly superior cellular uptake capacity and protease stability compared to linear peptides [46,47]. This chemical system exhibits excellent chemoselectivity and biorthogonality under aqueous conditions compared with the Sₙ2 reaction of alkyl halides and can be seamlessly adapted to HTS platforms such as phage display [48]. The chemical conjugation involving the *S*_N_Ar reaction between cysteine thiols and these perfluoro-linkers is illustrated in Figure 3.

Recently, the research focus in this field has shifted to functional applications. Through 3D blood–brain barrier (BBB) sphere model penetration depth tests and in vivo biodistribution experiments in mice, the status of perfluoroaryl (ArF) linkers as “BBB shuttling carriers” has been established, with a penetration efficiency 8–12 times that of ordinary alkyl linkers, and it can successfully deliver macromolecular substances such as antisense oligonucleotides [49]. The latest research by Peng et al. further revealed that perfluoro linkers can drive peptides to accumulate in lipid raft microdomains of the cell membrane, thereby triggering caveolin-mediated transcytosis (CMT) [50]. This transport pathway is significantly upregulated in the aging brain due to a 44.2% decrease in pericyte coverage and a 51.3% decrease in Mfsd2a expression. Experimental data show that the optimized perfluoroalkyl-stapled peptide has a cellular uptake intensity in brain endothelial cells 7.73 times that of linear peptides, and its BBB crossing efficiency in the aged model is 1.4 times higher than that in the young model. Perfluoroaryl linkers still face safety challenges: rigid ArF is prone to causing hemolysis and cytotoxicity, while flexible perfluoroalkyl (AlkF) can maintain the steady state of cell membrane fluidity with significantly better biosafety. Therefore, flexible fluoro chains have become the future development direction of this field.

#### 2.1.3. Bipyridine Linkers: Heteroatom-Directed IMHB Shielding

Although the hydrophobic scaffold of aromatic linkers offers certain advantages for improving membrane permeability, the extent of enhancement is often limited. In practical research and development, tedious *N*-methylation modifications are still commonly required to mask the backbone polarity, which significantly increases synthetic complexity and screening costs. In 2023, the Suga group proposed the use of bipyridine (BPy) as a macrocyclization unit for macrocyclic peptides (Figure 2d), providing a new approach to address this challenge [51].

Unlike traditional peptide stapling that relies solely on spontaneous backbone-driven IMHBs, the BPy unit is designed to play an active dual role. In addition to serving as a hydrophobic entity, it utilizes the pyridine nitrogen atoms as potent exogenous hydrogen-bond acceptors to directly coordinate and form IMHBs with the exposed NH donors on the opposite side of the peptide chain. This specific heteroatom-directed coordination effectively templates an IMHB network, shielding the polar backbone and significantly improving transmembrane efficiency without necessitating an excessive increase in global lipophilicity. Quantitative evaluation via the chloroalkane penetration assay (CAPA) showed that the transmembrane efficiency of model peptides incorporating BPy units was approximately 40-fold higher than that of the control group containing standard acetyl thioether bonds; in uncharged sequences, BPy also exhibited a 4.6-fold improvement compared to biphenyl (BPh) linkers [52]. Its membrane permeability even outperforms classic polycationic cell-penetrating peptides (CPPs) such as Tat and R9. Experimental results further confirmed that this performance is less affected by cyclization modes (e.g., macrocyclic or lariat structures) and peptide chain length, demonstrating exceptional structural robustness. The BPy linker has been verified as a dominant factor driving the transmembrane transport of macrocyclic peptides, laying an important foundation for the subsequent development of other high-affinity heterocyclic macrocyclic peptide drugs targeting intracellular targets.

### 2.2. Heteroaromatic Linkers Constructed from Unnatural Amino Acids (UAAs)

**Figure 4:**
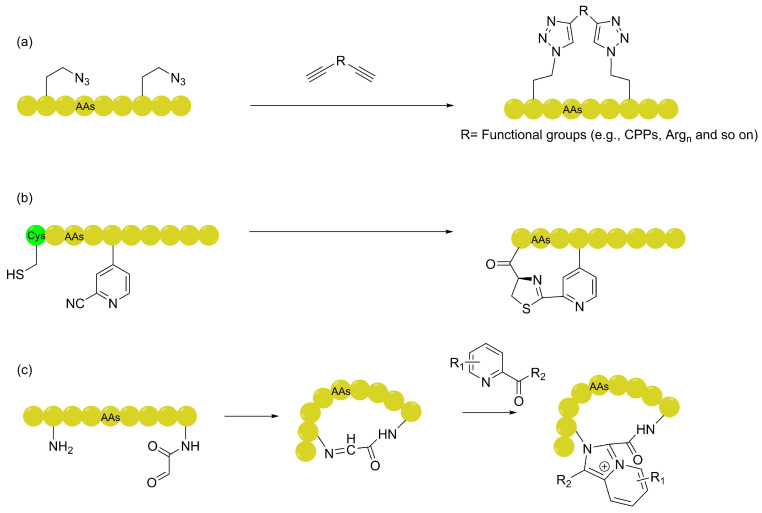
Representative heteroaromatic linkers derived from UAAs for cell-permeable macrocyclic peptides: (**a**) Triazole linkers featuring a modular R group (e.g., CPPs or cationic clusters) to enable a “decoupling strategy” for independent optimization of permeability and affinity. (**b**) Thiazoline linkers acting as rigid bending templates to facilitate localized IMHB networks, effectively reducing the polar surface area (PSA). (**c**) Imidazopyridinium (IP^+^) scaffolds enhancing passive diffusion via charge-hydrophobicity synergy, where the permanent cation drives membrane enrichment and the hydrophobic fused ring facilitates lipid core translocation.

Accompanied by the continuous enrichment of the chemical biology toolkit, a series of spontaneous, efficient, and biocompatible cyclization linkers has emerged. Beyond the aryl and polyfluoroaryl linkers widely employed in discovery stages, strategies capable of spontaneous cyclization under biocompatible conditions have garnered significant attention due to their exceptional capacity for physicochemical modulation [53,54]. These strategies typically circumvent the need for exogenous metal catalysts, not only simplifying the construction of high-throughput libraries but also reshaping the transmembrane properties of macrocyclic peptides at a fundamental molecular level through their unique heterocyclic architectures. Representative architectures of these heteroaromatic linkers, including triazole, thiazoline, and imidazopyridinium (IP^+^) scaffolds, are illustrated in Figure 4.

#### 2.2.1. Triazole Linkers: From Structural Stabilization to Decoupled Functionalization

Triazole-based linkers constructed via click chemistry have emerged as a pivotal strategy in both stapled peptide synthesis and mRNA display screening—facilitating, for instance, the direct competitive comparison of linear, monocyclic, and bicyclic libraries—owing to their exceptional reaction kinetics and high biorthogonality [55]. While simple triazole linkers can significantly enhance the helicity and proteolytic stability of peptides, their direct contribution to membrane permeability is often modest [47,56,57]. Nevertheless, the robust bioorthogonality of these linkers provides a versatile platform for functional modification to overcome this limitation. For instance, the conjugation of therapeutic peptides with membrane-permeable motifs, such as CPPs, has become a prominent approach for enhancing cellular uptake (enhancing the cell permeability of stapled peptides with a cyclic cell-penetrating peptide). Lau et al. have conducted a series of landmark studies in the field of triazole-linked two-component stapled peptides. By leveraging the efficient kinetics and high bioorthogonality of click chemistry, the team proposed an innovative “decoupling” strategy. This approach enables the independent optimization of membrane permeability and backbone binding affinity through the endogenous incorporation of cationic residues (e.g., Arg), the exogenous conjugation of cell-penetrating peptide sequences, or the utilization of diverse crosslinking toolboxes on the dialkynyl linkers. Confocal microscopy and reporter gene assays confirmed that these functionalized linker modifications significantly enhance cellular uptake efficiency. Notably, this strategy transcends the traditional dependence of stapling technologies on α-helical conformations, extending their application to non-helical/extended peptides (such as TNKS inhibitors) and significantly broadening the therapeutic potential of peptides for challenging intracellular targets [58,59,60].

#### 2.2.2. Thiazoline Linkers: Environment-Dependent IMHBs via Rigid Bending Templates

Inspired by the biosynthetic pathway of firefly luciferin, the thiazoline-bridged cyclization strategy has emerged as a highly efficient, “in-translation” approach compatible with high-throughput mRNA display platforms. Developed by the Hohsaka group, this strategy centers on an exceptionally mild and spontaneous condensation reaction between the N-terminal cysteine (Cys) and a side-chain nitrile group under physiological pH. Such spontaneous cyclization not only ensures the structural diversity of screening libraries but also, due to its excellent chemical orthogonality, provides robust technical support for the de novo selection of macrocyclic leads with intracellular targeting potential within mRNA display systems [61]. Experimental validation and physicochemical analyses have demonstrated that thiazoline-bridged peptides exhibit significantly superior membrane permeability compared to amide- or thioether-linked analogs of similar sequences. Tamura et al. confirmed via parallel artificial membrane permeability assays (PAMPAs) that thiazoline-based macrocycles achieve a substantial increase in apparent permeability (*P*_app_), even when compared to amide controls with higher or similar lipophilicity (*c* Log *P*/Log *D*). Rather than directly engaging in hydrogen bonding like the BPy linker, the thiazoline ring facilitates membrane permeability through a distinct structural mechanism. The biophysical mechanisms underlying this enhancement are two-fold: first, the spontaneous cyclization between the N-terminal cysteine and the side-chain nitrile group converts a hydrogen-bond donor (HBD) into an acceptor, directly reducing the overall molecular polarity. Second, temperature-variable NMR and computational simulations revealed that the rigid thiazoline scaffold serves as a rigid bending template, which strongly drives the peptide backbone to form transannular IMHBs exclusively in hydrophobic environments. This structural pre-organization results in a profound polar offset and spatial hydrophobic shielding effect by neighboring side chains, effectively compressing the PSA, thereby lowering the desolvation penalty during transmembrane translocation [62]. Crucially, this linker enables environment-dependent conformational adaptation: while these IMHBs effectively mask polarity in lipidic membranes, they can spontaneously dissociate in highly polar aqueous solutions to expose polar groups, ensuring sufficient water solubility.

#### 2.2.3. Imidazopyridinium (IP^+^): Challenging Passive Diffusion Dogmas with Charge-Hydrophobicity Synergy

Beyond the neutral linkers discussed above, the IP^+^ scaffold, as reported by Li et al., represents a significant advancement in macrocyclic permeability, fundamentally challenging the long-held dogma that charged moieties inherently preclude passive diffusion [63]. This unique linker overcomes the membrane barrier through a “charge-hydrophobicity synergy”: its permanent cationic center facilitates electrostatic enrichment at the anionic membrane surface, while the hydrophobic fused-ring system enables the molecule to “slide” through the lipid core as a pseudo-hydrophobic species. Validated by PAMPAs and CAPAs, IP^+^ linkers significantly augment the passive diffusion and cytosolic entry of high-molecular-weight macrocycles, effectively bypassing the endosomal entrapment common in traditional arginine-rich peptides. Furthermore, the IP^+^ macrocyclization exhibits exceptional chemical robustness and modularity—proceeding efficiently on miniaturized solid-phase resins with typical purities exceeding 85%—rendering it highly compatible with combinatorial library synthesis and holding great potential for DNA-encoded libraries (DELs). This practical utility was exemplified by the parallel solid-phase synthesis and HTS of a 480-member IP^+^-linked library, which yielded potent ligands with micromolar affinity, underscoring the platform’s potential for the de novo discovery of cell-permeable leads against challenging intracellular targets.

In recent years, the expansion of synthetic methodologies and chemical biology toolboxes has led to the emergence of novel linkers featuring aromatic or heterocyclic scaffolds [64,65,66,67,68,69]. Through the ongoing optimization of bioorthogonal reactivity and biocompatibility, these linkers are being progressively integrated into high-throughput platforms such as mRNA display and DELs. Their vast chemical diversity allows researchers to achieve the “front-loading” of favorable physicochemical traits during the earliest stages of selection. This shift from simple structural stabilization toward functional-driven design not only enhances the intrinsic quality of libraries but also provides a robust technical foundation for identifying cell-permeable leads with drug-like potential from trillion-scale chemical spaces.

## 3. Post-Screening Structural Refinement and Optimization Strategies

The bioactive sequences identified through high-throughput screening represent a starting point rather than a clinical finality. While display technologies excel at rapidly identifying high-affinity ligands, rational chemical refinement remains essential for addressing systemic hurdles, particularly membrane permeability. Linker engineering has evolved from basic structural bridging into a sophisticated strategy for precise permeability optimization [70]. In recent years, various functionalized linkers have demonstrated significant potential in facilitating cellular uptake. This section systematically reviews several representative linker strategies, focusing on their underlying mechanisms and recent breakthroughs in enhancing membrane permeability.

### 3.1. All-Hydrocarbon Staples: Refining the “Gold Standard” from Helical Stabilization to Metabolic Trapping

As the most established and widely used class of macrocyclic peptides, all-hydrocarbon stapled peptides have witnessed remarkable technological advancements to date, with their foundational research dating back to the late 1990s. Grubbs and Blackwell first utilized ruthenium-catalyzed ring-closing metathesis (RCM) to achieve side-chain crosslinking, establishing the chemical groundwork for the field [22,35,71]. In 2004, a landmark study by Walensky and Verdine published in *Science* demonstrated for the first time that all-hydrocarbon modification not only stabilizes the α-helical conformation but also confers exceptional cell-penetrating capabilities and induces apoptosis in vivo. This milestone marked the formal transition of stapled peptides into the era of intracellular therapeutic development [32].

In the ongoing evolution of the field, traditional design principles for membrane permeability—centered on lipophilicity, helicity, and charge distribution—are being continuously refined. Regarding the enhancement of peptide membrane permeability, the prevailing view, supported by numerous preceding studies, maintains that high lipophilicity and helicity promote cellular uptake by creating a “hydrophobic patch” and shielding the peptide backbone, thereby significantly reducing the desolvation energy penalty during translocation. While traditional perspectives held that positive charges were essential for initial electrostatic interactions with negatively charged cell membranes, and negative charges were viewed as barriers to entry, recent research has challenged these long-standing assumptions. Notably, in a landmark 2024 study, the Partridge, Brown, and Suga groups provided a robust revision of stapled peptide design rules based on a systematic analysis of over 350 variants [23]. To rigorously differentiate genuine cytosolic delivery from artifacts caused by membrane lysis, the researchers employed a comprehensive assay suite: the Nano-Click permeability assay for target-agnostic quantification, the LDH release assay for toxicity screening, and the Cell Ratio (*EC*_50_/*K_D_*) to evaluate translocation efficiency. Their data revealed a distinct “solubility cliff” for lipophilicity at Log *D* > 3 and a “threshold effect” for helicity (approximately 31%); furthermore, the strategic incorporation of anionic residues (glutamic acid) rather than cationic residues proved critical for balancing solubility and safety. Most significantly, they unveiled a “metabolic-driven intracellular retention” mechanism: a hydrophobic poly-alanine (Poly-Ala) tail facilitates initial passive entry, followed by intracellular enzymatic cleavage to generate a negatively charged “trapped” metabolite, thereby achieving efficient cytosolic accumulation. This integration of quantitative design rules and metabolic orchestration marks a paradigm shift in the development of high-performance, cell-permeable peptide therapeutics.

**Figure 5:**
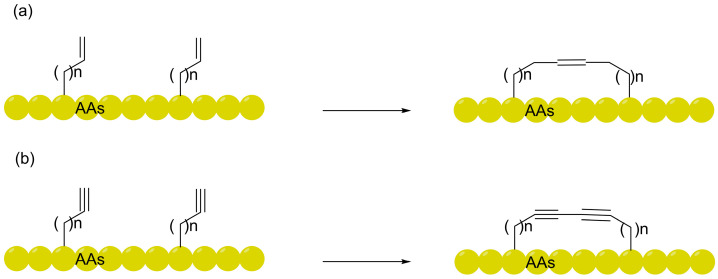
Representative all-hydrocarbon linkers for peptide stapling: (**a**) alkene-based staples generated via ring-closing metathesis (RCM); (**b**) alkyne-based linkers (diyne–girder) formed through oxidative coupling.

Besides, hydrocarbon linkers have undergone a notable evolution, shifting from simple sequence stabilization to the sophisticated orchestration of “advanced structural folding.” Ma et al. (2024) introduced a heteroconformational double-stapled peptide (DSARTC), which, for the first time, simultaneously stabilizes both α-helix and *β*-sheet motifs within a single molecule [72]. This dual-stapling strategy leads to a highly compact conformation that physically shields the polar peptide backbone, thereby significantly reducing the desolvation energy penalty during membrane translocation. Experimental evidence demonstrated that DSARTC’s permeability—validated by confocal microscopy and flow cytometry—surpassed that of traditional poly-arginine (PolyR) conjugation, transitioning the drug’s activity from ineffective to the micromolar range (*IC*_50_ = 4.6 μM) against AR-V7. This highlights that structural pre-organization is often superior to simple cationic tagging for driving cellular uptake. Simultaneously, chemical innovations in linker architecture have provided non-destructive modalities for monitoring permeability. The “diyne–girder” technology developed by Jamieson et al. utilizes Glaser oxidative coupling to yield rigid, single-isomer linkers [73,74]. Beyond inducing high helicity (up to 59%) to minimize entropic loss, these linkers exhibit a strong Raman signal at 2252 cm^−1^ within the “cell-silent region.” This allows for the direct tracking of peptide intracellular distribution via Raman microscopy without the interference of bulky fluorophores. Such bifunctional linkers not only enhance subtype selectivity (e.g., a 100-fold preference for MDM2 over MDMX) through extreme rigidity but also offer a unique tool for observing “authentic” peptide transport. The chemical structures of representative all-hydrocarbon linkers, including alkene and alkyne motifs, are shown in Figure 5.

### 3.2. Fluorine–Thiol Displacement Reaction (FTDR): Global Coil-to-Helix Chameleonicity

**Figure 6:**
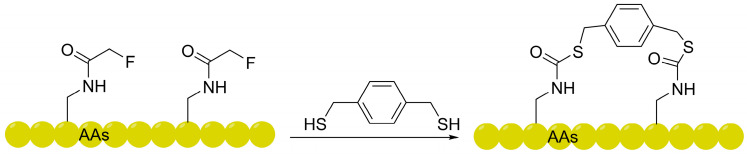
FTDR-mediated stapling using aromatic dithiols. This strategy leverages “chameleonic adaptability” to shield polar surfaces through environment-induced helicity shifts.

The fluorine–thiol displacement reaction (FTDR) stapling technology, developed by the Wang and Yang groups, offers a sophisticated chemical modality to surpass the permeability constraints of traditional hydrocarbon staples [75,76]. The conjugation chemistry and the resulting scaffold of FTDR-mediated linkers are illustrated in Figure 6. While heteroaromatic linkers (such as thiazoline) achieve conformational adaptation through the solvent-dependent regulation of localized IMHB networks, the “chameleonic adaptability” mediated by FTDR aromatic dithiol linkers represents an extreme form of this property, achieving dynamic shielding via a global secondary structure transition. In silico modeling and environmental studies demonstrate that FTDR peptides maintain innate flexibility in aqueous media but undergo a dramatic conformational shift upon membrane contact, with α-helicity surging from ~12% to ~89%. This extensive coil-to-helix transition enables an extraordinary Δ-PSA between water and lipid-like environments, effectively masking the PSA dynamically during translocation. To rigorously quantify this uptake, researchers employed confocal microscopy and flow cytometry, utilizing Trypan Blue quenching to eliminate extracellular background and confirm genuine cytosolic internalization. Quantitative analysis revealed that FTDR-stapled peptides achieve up to a 5-fold increase in cellular uptake compared to RCM-based analogs. Mechanistic elucidation through inhibitor panels (e.g., chlorpromazine, nystatin) and competition assays indicated that the FTDR scaffold bypasses the predominantly heparan sulfate proteoglycan (HSPG)-dependent pathway characteristic of hydrocarbon staples. Instead, it recruits multiple endocytic machineries, including clathrin-, caveolin-, and actin-mediated pathways, particularly when optimized with an L,D-stereochemical configuration that facilitates nuclear enrichment. This synergy between multi-channel endocytosis and dynamic conformational shielding translates into potent intracellular inhibition of challenging targets like the Wnt signaling pathway and ERα-SRC2.

### 3.3. Self-Tracing Linkers: Eliminating Experimental Artifacts via Intrinsic Fluorescence

**Figure 7:**
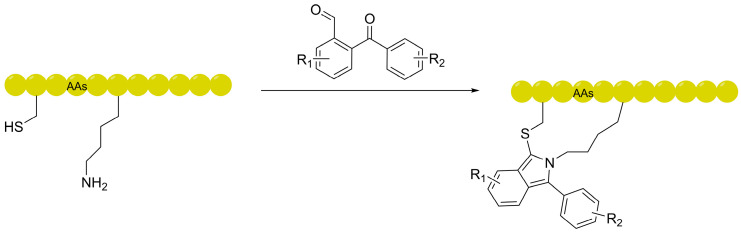
Schematic representation of the fluorescent isoindole crosslinking (FlICk) strategy. The reaction between ortho-phthalaldehyde (OPA) and the side chains of lysine and cysteine residues forms a fluorescent isoindole bridge at the *i*, *i* + 4 positions, providing both structural stabilization and intrinsic self-tracing functionality.

Beyond the previously mentioned strategies of utilizing diyne groups for Raman spectroscopy detection (located in the cell silent region) to achieve non-destructive observation, chemical innovations in linkers have also catalyzed the development of molecular tools with intrinsic autofluorescent properties. The fluorescent isoindole crosslinking (FlICk) technology, developed by the research groups of Perrin, Li, and Chen, provides a more intuitive “what you see is what you get” solution. This technique employs ortho-phthalaldehyde (OPA) or its derivatives (ArKBCHOs) to undergo site-specific conjugation with lysine and cysteine residues on peptide side chains [77]. The site-specific conjugation chemistry and the resulting isoindole architecture are illustrated in Figure 7. This process constructs a characteristic fluorescent isoindole bridge in situ at the *i*, *i* + 4 positions. The core advantage of this design is that it effectively mitigates the potential for the “experimental artifact” problem common in traditional membrane permeability studies—where the mandatory attachment of bulky hydrophobic dyes (such as FITC) can bias results. Consequently, FlICk provides more intrinsically representative intracellular transport data by minimizing perturbations from exogenous bulky fluorophores. Experimental evidence confirms that FlICk-stapled peptides exhibit significant intracellular accumulation in DLD-1 and Jurkat. The underlying mechanism is that the FlICk linkage—whose atom count perfectly matches that of typical olefinic staples—effectively enforces an α-helical conformation. These linkers, which combine structural constraint with self-tracing functionality, not only replicate the biological activity of traditional hydrocarbon-stapled peptides but also—due to their mild reaction conditions and potential for HTS—offer a precise chemical platform for the development of theranostic peptide drugs.

### 3.4. Stimuli-Responsive Reversible Staples: Harmonizing Permeability with Pharmacophore Regeneration

To navigate the delicate balance between membrane permeability and native bioactivity, recent linker engineering has explored both permanently charged scaffolds and environment-responsive reversible “unstapling” strategies. Li, Yin, and colleagues developed a highly efficient methionine bis-alkylation strategy that incorporates dual sulfonium cations into the linker. When applied to target the hepatitis B virus (HBV) HBx/Bcl-2 protein–protein interaction, this permanently charged linkage significantly enhanced electrostatic enrichment and cellular uptake [78]. In this scenario, the stapled scaffold directly acts as the active entity, improving the binding affinity to Bcl-2 from >45.6 μM (linear) to 5.3 μM and yielding potent antiviral effects without significant cytotoxicity [79].

**Figure 8:**
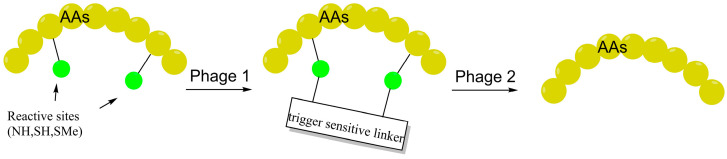
General mechanism of environment-responsive reversible stapling: Phase I: In vitro peptide locking via site-specific conjugation with trigger-sensitive linkers. Phase II: Stimuli-driven (e.g., GSH, H_2_O_2_) intracellular “unstapling.” This decoupling strategy enables traceless pharmacophore regeneration and reconciles the trade-off between membrane permeability and target affinity.

Building upon the chemical reversibility of specific linkers to act as true intracellular prodrugs, Wan and coworkers introduced a carbamate-based “one-trigger, two-releases” platform leveraging a dual 1,4-elimination mechanism responsive to GSH or H_2_O_2_ [80]. In the design of LSD1 inhibitors, although stapling weakened in vitro *Ki* values by 16–36 fold, 4T1 cell viability assays demonstrated superior potency over the linear parent, underscoring the synergy between enhanced passive permeability and intracellular release. Similarly, Qian et al. developed reversible bicyclic scaffolds using disulfide bonds to achieve stimulus-responsive cargo release. Validated by flow cytometry and dual-luciferase reporter assays against the NEMO-IKK interaction, this system exhibited 3-fold higher uptake than standard monocyclic peptides and extended the serum half-life to 10 h [81]. By integrating charge-induced uptake, passive diffusion, and environment-activated release, these dynamic linkers offer intelligent solutions for the precision targeting of intracellular proteins (Figure 8).

Beyond the aforementioned mainstream strategies, several novel linker architectures have recently emerged, including bis-urea bridges, double-guanidinium crosslinks, and foldamer-4MP hybrid topologies [82,83,84]. Although these structures possess diverse chemical natures, their core design principles are highly consistent: they aim to lower the transmembrane energy barrier through conformational rigidification, polar shielding, charge modulation, or environmental responsiveness. However, these cases also serve as a cautionary tale; blind charge accumulation or excessive lipophilicity often carries a risk of hemolytic toxicity or endosomal trapping. This further underscores the urgency of transitioning evaluation frameworks from “apparent cellular entry” toward the establishment of more physiologically relevant platforms for “effective cytosolic quantification”.

Notably, the demarcation between “biocompatible post-modification” and “HTS-compatible chemistry” is becoming increasingly blurred. For instance, ortho-phthalaldehyde (OPA)-based FlICk chemistry, originally viewed as a specific tool for rational structural modification, is currently being explored for broader genetic encoding platforms due to its exceptional reaction efficiency and in situ fluorogenic properties [85]. Similarly, ruthenium-catalyzed RCM for all-hydrocarbon stapling—once considered unsuitable for aqueous high-throughput screening—has recently achieved significant breakthroughs in its compatibility with DELs [86,87,88]. This trend suggests that linker selection is no longer a binary opposition between “discovery screening” and “rational design.” Instead, it is an integrated process that deeply unifies high-capacity screening capabilities with complex chemical functionalities, aimed at collectively conquering the frontier of intracellular targeting.

## 4. Discussion

### 4.1. Evolution of Evaluation Frameworks: From Apparent Uptake to Effective Cytosolic Quantification

The evaluation framework for stapled peptide membrane permeability has undergone a paradigmatic leap from “apparent cellular uptake observation” to “effective cytosolic quantification.” Early research exhibited an over-reliance on confocal laser scanning microscopy and flow cytometry—paradigms frequently prone to membrane enrichment-induced false positives. These errors often stem from the hydrophobic synergistic effects between linkers and exogenous fluorescent probes (such as FITC), which can lead to an overestimation of a molecule’s true permeability potential.

As the field matures, evaluation tools are evolving into integrated platforms with higher physiological relevance. While PAMPA facilitates the high-throughput screening of passive diffusion potential and effectively captures the physical benefits of conformational pre-organization, its nature as an artificial lipid bilayer precludes it from accounting for active transport mechanisms (e.g., caveolae-mediated endocytosis) or complex biological barriers such as endosomal entrapment and efflux pumps. Consequently, target-agnostic intracellular assays—such as the chloroalkane penetration assay (CAPA) and Nano-Click technology—coupled with membrane integrity evaluations (e.g., LDH release assays) have emerged as the new gold standard. This orthogonal approach enables researchers to precisely pinpoint “effective cytosolic concentration” while rigorously ruling out artifacts caused by membrane lysis. At the heart of this transition lies a profound understanding of the complex, non-linear relationship between physicochemical properties and biological outcomes. The structural features, mechanisms, and evaluation methods of the various linkers discussed in this review are summarized in Table 1.

### 4.2. Helicity-Driven Permeation Mechanisms and Systematic Physicochemical Trade-Offs

For decades, increasing helicity, lipophilicity, and positive charge density have been regarded as the “golden rules” for enhancing peptide membrane permeability. For instance, classical all-hydrocarbon linkers lock peptides into highly α-helical conformations, forcing the backbone amides into dense IMHB networks, which significantly mitigates the desolvation energy penalty during transmembrane translocation. Furthermore, helical stabilization necessitates the outward exposure of non-polar side chains, which, together with the linker, form “hydrophobic patches” that directly drive the partitioning of the peptide into the lipid core of the bilayer. However, all-hydrocarbon stapling via RCM relies on expensive transition-metal catalysts and exhibits limited functional group tolerance (e.g., incompatibility with unprotected thioureas). As more synthetically accessible linkers emerge, the correlation between these traditional metrics and permeability is increasingly being challenged. Simply increasing helicity does not always yield superior permeability, and blindly pursuing high lipophilicity or net charge can lead to severe adverse effects. First, when lipophilicity exceeds a threshold (e.g., Log *D* > 3), peptides frequently encounter a “solubility cliff,” resulting in formulation challenges and impaired systemic bioavailability. Extreme lipophilicity not only induces β-sheet-mediated aggregation but also promotes non-specific binding to serum proteins (such as HSA), which can lead to a significant loss (e.g., >10-fold) of cellular potency in serum-rich environments. At the biophysical level, highly rigid linkers, such as perfluoroaromatics (ArF), leverage the “fluorine effect” to promote partitioning; however, their planar, rigid scaffolds can perturb the lipid bilayer arrangement, triggering hemolytic toxicity and LDH release. Second, an excessive net positive charge can trigger electrostatic cytotoxicity and cause endosomal entrapment due to overly tight interactions with cell-surface proteoglycans. When extreme hydrophobicity is coupled with a high positive charge, it further elevates the risk of severe mast cell degranulation (MCD) and systemic toxicity. Consequently, modern design paradigms are shifting toward “chameleonic” strategies and “negative charge safety design,” or employing decoupling strategies such as stimuli-responsive reversible stapling to separate delivery functions from target binding. These diverse linker strategies and their associated physicochemical trade-offs are systematically summarized in Table 2. By orchestrating these parameters, researchers aim to resolve the long-standing trade-off between solubility, permeability, and safety.

### 4.3. Recent Advances in Computational Modeling and Machine Learning for Permeability Prediction

While traditional empirical descriptors have long guided peptide optimization, recent breakthroughs in computational modeling and machine learning offer a promising paradigm for predicting and enhancing membrane permeability. Seminal studies by the Jacobson group demonstrated that integrating 3D conformational flexibility and physics-based desolvation models significantly outperforms conventional 2D metrics for complex peptidic molecules [89,90]. Complementing these predictive frameworks, computational tools have enabled the de novo design of transmembrane proteins, as pioneered by the DeGrado group using the CHAMP algorithm to target lipid-embedded receptors with customized topologies [91].

Building upon these foundational physical and structural insights, the field is currently witnessing a surge in sophisticated deep learning models that address the unique topological challenges of macrocycles. Advanced frameworks now move beyond simple whole-molecule descriptors to capture multi-scale interactions; for instance, CycPeptMP integrates multi-level molecular features spanning from the atomic to the full-peptide level [92]. MultiCycPermea successfully fuses 2D structural images with 1D sequence information [93]. Furthermore, state-of-the-art platforms like PEGASUS are overcoming the historical scarcity of permeability data by combining massively parallel proxy biological assays with quantum mechanical (QM) simulations [94]. Looking forward, the synergistic integration of 3D conformational physics, high-throughput assay data, and topologically aware deep learning architectures will establish increasingly robust predictive tools, potentially streamlining the rational design of membrane-permeable peptide therapeutics.

## 5. Conclusions

This review systematically delineates the logical trajectory of stapled peptide linkers designed to enhance transmembrane permeability, tracing their evolution from early empirical all-hydrocarbon modifications to modern HTS and precision chemical entities. Supported by standardized evaluation frameworks, the academic understanding of “chameleonicity” and the mechanistic balance between charge and lipophilic thresholds has shifted from qualitative observation to quantitative analysis, substantially mitigating the experimental artifacts prevalent in earlier studies.

While current drug discovery and development efforts are still dominated by chemical modification strategies, stapled peptide design is undergoing a notable shift from “empirical trial-and-error” toward a “rational data-driven” approach. In the future, by integrating high-throughput biological data generated via mRNA display and leveraging artificial intelligence (AI) and machine learning for deep mining of multidimensional chemical spaces, we anticipate facilitating the move toward automated prediction of linker performance and multidimensional synergistic optimization. This interdisciplinary fusion will offer strategic insights to mitigate the permeability and bioavailability bottlenecks hindering the clinical translation of stapled peptides, laying a robust foundation for the development of innovative drugs targeting challenging, intracellular “undruggable” targets.

## Figures and Tables

**Table 1:** Summary of representative linker engineering strategies, their underlying membrane permeation mechanisms, and corresponding evaluation methodologies for stapled peptides.

Linker Strategy & Examples	Primary Permeation Mechanism	Key Evaluation Assays	Refs.
**Arylene-based** (mDBMB, TBMB, BPh)	Enhances permeability via conformational rigidification; “topological condensation” reduces the molecular radius of gyration and entropic penalty.	FITC imaging, CETSA, live-cell imaging.	[36,40]
**Perfluoro-aromatic** (ArF, AlkF)	Leverages the “Fluorine Effect” to increase lipophilicity; drives lipid raft accumulation and triggers CMT.	3D BBB sphere model, flow cytometry, confocal microscopy.	[46,47,49,50]
**Bipyridine (BPy)** (BPy unit)	Pyridine nitrogens act as H-bond acceptors to form IMHBs with backbone amides, providing effective shielding and increasing lipophilicity.	CAPA, PAMPA.	[51]
**Triazole-based** (dialkynyl linkers)	Employs a “decoupling” strategy to independently optimize permeability (via cationic motifs or CPPs) and binding affinity.	Confocal microscopy, reporter gene assays.	[47,56,57]
**Thiazoline** (cys-nitrile condensation)	Converts an HBD into an HBA; induces a stable IMHB network in hydrophobic environments to compress the PSA and lower desolvation energy.	PAMPA, temp-variable NMR, Computational simulations.	[62]
**Imidazo-pyridinium (IP^+^ scaffold)**	Overcomes the membrane barrier via charge-hydrophobicity synergy, where the permanent cation facilitates membrane enrichment while the fused-ring system enables “pseudo-hydrophobic sliding”.	PAMPA, CAPA.	[63]
**All-hydrocarbon** (RCM-based staples)	Stabilizes α-helical structures to shield polar amides; utilizes metabolic-driven trapping where enzymatic cleavage of a Poly-Ala tail leads to cytosolic accumulation.	Nano-Click, LDH release, cell ratio (*EC*_50_/*K_D_*).	[23,72,73,74]
**FTDR-based** (aromatic dithiols)	Exhibits “chameleonic adaptability” with dynamic helicity increases (from 12% to 89%) upon membrane contact; bypasses HSPG-dependence via multiple endocytic pathways.	Flow cytometry, Trypan Blue quenching, inhibitor panels.	[75,76]
**Self-tracing** (isoindole, diyne)	Mitigates artifacts from external dyes using intrinsic fluorescence (FlICk) or Raman signals in the “cell-silent” region.	Raman microscopy, live-cell fluorescence imaging.	[77]
**Stimuli-responsive** (sulfonium, carbamate)	Utilizes permanently charged scaffolds for direct targeting or employs intracellular “unstapling” (e.g., GSH/H_2_O_2_ triggered) to tracelessly regenerate the pharmacophore.	MFI, co-localization, flow cytometry.	[78,79,80,81]

**Abbreviations:** BBB, blood–brain barrier; CAPA, chloroalkane penetration assay; CETSA, cellular thermal shift assay; CMT, caveolin-mediated transcytosis; CPP, cell-penetrating peptide; FITC, fluorescein isothiocyanate; GSH, glutathione; HBA, hydrogen-bond acceptor; HBD, hydrogen-bond donor; HSPG, heparan sulfate proteoglycan; IMHB, intramolecular hydrogen bond; LDH, lactate dehydrogenase; MFI, mean fluorescence intensity; PAMPA, parallel artificial membrane permeability assay; PSA, polar surface area.

**Table 2:** Summary of synthetic complexity, core advantages, and physicochemical trade-offs of linker strategies.

Linker Strategy & Examples	Synthetic Complexity	Core Advantages	Limitations & Trade-Offs (Toxicity/Biocompatibility)
**Arylene-based** (*mDBMB*, *TBMB*)	**Low**: Spontaneous reaction in aqueous media; catalyst-free.	**HTS Compatibility**: Significantly compresses the radius of gyration; ideal for large-scale library screening.	**Performance Ceiling**: Rigidity alone provides a limited contribution to permeability; high aromaticity poses solubility risks.
**Perfluoroaromatic** (*ArF*)	**Low**: Metal-free with high chemoselectivity.	**Enhanced Lipophilicity:** Utilizes the “Fluorine Effect” to increase global hydrophobicity and facilitate passive membrane translocation.	**Hemolysis Risk**: Rigid scaffolds may perturb membrane fluidity; exhibits dose-dependent toxicity.
**Bipyridine** (*BPy*)	**Medium**: Requires specific building blocks but offers high cyclization efficiency.	**Polar Shielding**: Acts as an exogenous acceptor to template **IMHB** networks without increasing global lipophilicity.	**Spatial Sensitivity**: Highly dependent on precise geometric orientation for effective hydrogen-bond shielding.
**Triazole-based** (dialkynyl linkers)	**Low**: Rapid kinetics and high bioorthogonality.	**Modular Decoupling**: Allows independent optimization of delivery motifs (e.g., CPPs) and binding affinity.	**Poor Passive Permeability**: The triazole ring itself contributes little to permeation; often requires auxiliary motifs.
**Thiazoline** (cys-nitrile condensation)	**Low**: Spontaneous “in-translation” condensation; suitable for mRNA display.	**In-situ Shielding**: Converts a donor (HBD) into an acceptor (HBA); compresses PSA in hydrophobic phases.	**Sequence Dependence**: Strictly limited by the specific spacing between the N-terminal Cys and the side-chain nitrile.
**Imidazo-pyridinium (IP^+^ scaffold)**	**Low**: Multi-component condensation compatible with solid-phase synthesis.	**Charge Synergy:** Leverages permanent charge and rigid hydrophobicity for pseudo-hydrophobic sliding.	**Bulky Scaffold:** Significantly increases overall molecular weight; the permanent cation may induce non-specific binding in complex serum environments.
**All-hydrocarbon** (RCM-based staples)	**High**: Dependent on expensive catalysts and anhydrous/oxygen-free conditions.	**Robust Helical Stabilization:** Highly effective at nucleating α-helices; fully compatible with advanced delivery modules	**Solubility Cliff**: Extreme lipophilicity (Log *D* > 3) may lead to aggregation and MCD risks.
**FTDR-based** (aromatic dithiols)	**Low**: Aqueous compatibility; yields isomer-free products with high efficiency.	**Conformational Adaptability**: Undergoes a significant helicity shift upon membrane contact.	**Stereochemical Preference**: Strong dependence on peptide chirality combinations (e.g., L,D-configurations).
**Self-tracing** (isoindole, diyne)	**Medium**: In-situ fluorescence generation; reduces dye interference.	**Intrinsic Traceability:** Mitigates artifacts in permeability assays caused by bulky external fluorophores.	**Structural Constraints**: Requires specific residue spacing (e.g., Cys-Lys) for bridge formation.
**Stimuli-responsive** (sulfonium, carbamate)	**Medium**: Requires introduction of environment-sensitive redox sites.	**Performance Coordination**: Resolves the conflict between “rigid permeation” and “flexible binding.”	**Release Kinetics**: Release rates must be precisely matched with intracellular metabolic windows and target affinity.

## Data Availability

No new data were created or analyzed in this study. Data sharing is not applicable to this article.

## Abbreviations

The following abbreviations are used in this manuscript:

PPI	Protein–protein interaction
HTS	High-throughput screening
HBDs	Hydrogen-bond donors
DEL	DNA-encoded library
ArF	Perfluoroaryl
AlkF	perfluoroalkyl
IMHB	Intramolecular hydrogen bond
PSA	Polar surface area
RCM	Ring-closing metathesis
CPPs	cell-penetrating peptides
CLIPS	Chemical Linkage of Peptides onto Scaffolds
TBMB	1,3,5-Tris(bromomethyl)benzene
BBB	Blood–brain barrier
BPy	Bipyridine
UAA	Unnatural amino acid
HSPG	Heparan sulfate proteoglycans
IP^+^	Imidazopyridinium
PAMPA	Parallel artificial membrane permeability assay
CAPA	Chloroalkane penetration assay
FTDR	Fluorine–thiol displacement reaction
OPA	ortho-phthalaldehyde
FlICk	Fluorescent isoindole crosslinking
QM	quantum mechanical
AI	Artificial intelligence
